# MiRNA target enrichment analysis of co-expression network modules reveals important miRNAs and their roles in breast cancer progression

**DOI:** 10.1515/jib-2022-0036

**Published:** 2024-12-25

**Authors:** Mohammad Javad Bazyari, Seyed Hamid Aghaee-Bakhtiari

**Affiliations:** Bioinformatics Research Center,Basic Sciences Research Institute, 37552 Mashhad University of Medical Sciences, Mashhad, Iran; Department of Medical Biotechnology and Nanotechnology, Faculty of Medicine, 37552 Mashhad University of Medical Sciences, Mashhad, Iran

**Keywords:** systems biology, gene expression regulation, functional enrichment analysis, protein-protein interaction network, molecular complex detection

## Abstract

Breast cancer has the highest incidence and is the fifth cause of death in cancers. Progression is one of the important features of breast cancer which makes it a life-threatening cancer. MicroRNAs are small RNA molecules that have pivotal roles in the regulation of gene expression and they control different properties in breast cancer such as progression. Recently, systems biology offers novel approaches to study complicated biological systems like miRNAs to find their regulatory roles. One of these approaches is analysis of weighted co-expression network in which genes with similar expression patterns are considered as a single module. Because the genes in one module have similar expression, it is rational to think the same regulatory elements such as miRNAs control their expression. Herein, we use WGCNA to find important modules related to breast cancer progression and use hypergeometric test to perform miRNA target enrichment analysis and find important miRNAs. Also, we use negative correlation between miRNA expression and modules as the second filter to ensure choosing the right candidate miRNAs regarding to important modules. We found hsa-mir-23b, hsa-let-7b and hsa-mir-30a are important miRNAs in breast cancer and also investigated their roles in breast cancer progression.

## Abbreviations

BP: biological process
CC: cellular compartment
FDR: false discovery rate
GO: gene ontology
GSVA: gene set variation analysis
KEGG: Kyoto encyclopedia of genes and genomes
MCODE: molecular complex detection
MF: molecular function
PPI: protein-protein interaction
TCGA: the cancer genome atlas
TOM: topology overlap measure
WGCNA: weighted co-expression network analysis

## 1 Introduction

Breast cancer is the most common cancer in the world and the fifth cause of death due to cancer. Among the women population, it accounts for 25 % of cancer incidence and 16.6 % of cancer mortality [1]. There are several risk factors associated with the development and progression of breast cancer. Factors such as familial history of breast cancer, history of chest radiation therapy and hormone replacement therapy can increase the chance of cancer development. Besides, genetic risk factors also play an important role in breast cancer. Inherited mutations in several genes such as CHEK2, CDH1, ATM, and TP53 are known to be breast cancer risk factors. Mutations in BRCA1 and BRCA2 are the most common cause of hereditary breast cancer and the most notable genetic risk factors [2]. Because of its heterogeneous nature, breast cancer cells can change instantly and adapt to their microenvironments. This leads to the tumor progression and metastasis which are associated to poor survival of patients [3]. There has been an improvement in the survival of patients because of screening and treatment strategies in breast cancer; but still median survival after metastasis or distant recurrence is 38 months [4].

miRNAs are small non coding RNA that play an important role in the regulation of gene expression. Mostly they have negative regulatory effects on their targets by interacting with the 3′ untranslated regions (3′ UTR) and inducing degradation [5]. Different phenomena in breast cancer such as progression, recurrence, metastasis and resistance to chemotherapy are influenced by these master regulators of gene expression. According to their effects on cancer-related pathways, there are two types of miRNAs: oncogenic miRNAs which are upregulated in cancer tissues and tumor suppressor miRNAs which are downregulated in cancer tissues [6]. For instance miR-200c and miR-141 are both oncogenic miRNAs in breast cancer and are associated with invasion and metastasis. On the other hand, miR-195 is a tumor suppressor miRNA and has anti-metastatic and anti-invasion effect on breast cancer cells [7].

Single miRNA can target multiple mRNAs and also the same gene can be targeted by several miRNAs [8], thus holistic approaches are needed to understand their regulatory effects. Systems biology which has been introduced two decades ago, offers various approaches to understand biological systems in more dynamic and holistic ways. High-throughput technologies like DNA sequencing and RNA sequencing make system-level understanding of biological phenomena more feasible and these system-level insights can be used for drug discovery [9]. Network representation and analysis is one of the popular approaches in systems biology. There are several types of biological networks such as metabolic network, protein-protein interaction (PPI) network, and gene regulatory network. These networks can be constructed based on existing knowledge or can be driven directly from the data and most of the time they are represented as ball-stick diagrams in which components are shown as nodes and their interactions as edges. Analysis of these biomolecular networks can help us to understand the design principles of biological systems and give us a clue about the rational behind them. For instance, scale-free topology, in which connectivity distribution fits a power law is the most common topology of different biological networks. Scale-free topology makes biological networks more robust to random mutation and failures, and also makes them small-world networks, in which there are highly connected clusters connected to each other [10].

Co-expression network is one of the biological networks which has been widely used in recent years. Co-expression networks are based on similarity scores between pairs of genes and their analysis can help to identify and cluster genes with similar patterns of expression. They can be used to understand the biological function(s) of correlated genes and also find a new set of genes that may have important roles in specific biological processes [11]. Co-expression networks are data-driven and they can reveal a different set of insights in comparison to other types of biological network such PPI networks which are based on existing knowledge. Although there can be an overlap between the co-expression network and gene-regulatory network of the same biological system [12]. One of the most successful tools for co-expression network construction and analysis is R package Weighted co-expression network analysis (WGCNA). It recruits different algorithms to find highly correlated genes and cluster them into a module [13].

Several studies have investigated important molecules and pathways in breast cancer by using WGCNA. For instance, WGCNA has been used in several studies to find new important biomarkers and modules which are related to the survival outcome of patients with breast cancer [14, 15]. Also WGCNA has been used to find important miRNAs and reveal their interaction with their targets by analyzing miRNA co-expression networks, mRNA co-expression networks, or both of them [16, 17]. One way to analyze and find important genes in co-expression networks that may gain little attention in breast cancer study, is enrichment analysis of co-expression network’s modules to understand their possible common regulators. Genes in one module have the same expression pattern and this similarity can be due to the regulatory effect of one or more regulators. In this study we aim to examine miRNAs by using systems biology approach to understand their system-level effects in breast cancer progression. To do so, first we use co-expression network of mRNAs to investigate breast cancer and find progression-related important gene modules. Then we examine possible regulatory effects of miRNAs on those modules by miRNA target enrichment analysis.

## 2 Materials and methods

### 2.1 Data source and pre-processing

RNA-seq, miRNA-seq, and clinical data of TCGA-BRCA project were downloaded by R package TCGAbiolinks [18], and imported to R programming environment. To eliminate possible effects of treatments on molecular mechanisms, those patients who have received any pharmaceutical or radiation therapy were removed from dataset. From those treatment-naive patients, tumor samples which have both RNA-seq and miRNA-seq data were chosen for further analysis. In addition, samples without stage information were removed. To filter out lowly expressed features, mRNAs and miRNAs which have less than 50 and 10 total counts number respectively among all samples were removed. Then sequencing data were normalized and log2 transformed. To find targets of miRNAs, Tarbase [19] and mirtarbase [20] which are both curated databases of mRNA-miRNA interactions were used. Both databases were merged into a single matrix and mRNAs that exist in the merged matrix were chosen and used to construct co-expression network. Before network construction, the quality of mRNA data was assessed by hierarchical clustering analysis.

### 2.2 Co-expression network construction

WGCNA [13], which is a powerful R package was used to construct signed gene co-expression network. To do so, first Pearson’s correlation coefficients between genes were calculated and used to construct adjacency matrix as follow:

$$a_{ij}={\left(\left|\left(1+\text{cor}\left(x_{i},x_{j}\right)\right)\right|/2\right)}^{\beta }$$

where *a* is adjacency matrix, cor(*x*_*i*_*, x*_*j*_) is Pearsion’s correlation coefficient between gene *i* and *j*, and *β* is soft threshold which eliminate weak correlation. To find appropriate soft threshold, expression matrix was transformed to similarity matrix and different values of *β* were tested by pickSoftThreshold function. Finally the smallest value which ensured constructed network has scale-free topology by *R*^2^ > 0.9 was chosen. Next, topology overlap measure (TOM) was calculated according to adjacency matrix and different gene modules have been distinguished by using dynamicTreeCut method for dendrogram of dissimilarity (1-TOM). Minimum required size of detected modules were set to 30 (minModuleSize = 30) and modules with less than 0.25 height in dendrogram were merged together (MergeCutHeight = 0.25). Other parameters of blockwiseModules function of WGCNA package, were set as follow: genes were split to blocks with maximum number of 5,000 (maxBlockSize = 5,000); Pearson was used as correlation type (corType = “pearson”); deepSplit = 2 was used for moderate sensitivity over module splitting; minimum connectivity of 0.5 between module’s eigengenes and 10 members was set as threshold to maintain detected modules (minCoreKME = 0.5, minCoreKMESize = 10); also module’s member with connectivity less than 0.3 to corresponding module’s eigengene were omitted from the module (minKMEtoStay = 0.3).

For each module, a representative value (eigengene) was calculated. To find important modules in cancer progression, correlations between module eigengenes and stage-related traits were calculated. Those modules that have |correlation| bigger than 0.2 and p-value less than 0.05 with at least two traits related to progression (clinical stage and TNM) were considered as important modules.

### 2.3 MiRNA target enrichment and correlation analysis

To test whether targets of specific miRNA are enriched in specific network modules, hypergeometric test was performed as follows:

$$P_{\text{HT}}=\overset{n}{\underset{k=z}{\sum }}\frac{\left(\begin{array}{c} x\\ k \end{array}\right)\left(\begin{array}{c} N-x\\ n-k \end{array}\right)}{\left(\begin{array}{c} N\\ n \end{array}\right)}$$

*P*_HT_: hypergeometric test *p*-value

*x*: the number of genes in a module

*n*: the number of targets of a specific miRNA

*z*: the number of module’s members and miRNA’s targets

*N*: total number of genes in the co-expression network

The total number of genes were 17,463 (*N* = 17,463) and the test was performed for 770 miRNAs against significant modules. Because multiple tests were performed, *p*-value was corrected by Benjamini–Hochberg method and False Discovery Rate (FDR) < 0.05 was chosen as the first threshold. As the second criterion, we used correlation between miRNAs and modules’ eigengenes. Here we observed Pearson’s correlation coefficients ranging from −0.56 to 0.89 and because miRNAs mostly have negative regulatory effect on gene expression, correlation less than −0.3 was set as the second threshold. Thus miRNA was flagged as significant if it had both significant number of targets in the specific module according to hypergeometric test result (FDR < 0.5) and negative correlation (coefficient < −0.3) with that module.

### 2.4 MiRNAs’ targeted genes GSVA score

Gene set variation analysis (GSVA) [21] is a popular and open-source R package and estimates variation of gene set in each sample of the population. For each significant miRNA, their target genes in targeted module were considered as a gene set and their GSVA score was calculated and compared among different stages of breast cancer.

### 2.5 Pathway and gene ontology enrichment analysis

DAVID database [22] and Cytoscape plugin, ClueGO [23] were used to performed functional enrichment analysis. When using DAVID, we chose three types of GO terms (biological process [BP], cellular compartment [CC], and molecular function [MF]) and KEGG (Kyoto Encyclopedia of Genes and Genomes) pathways as resources. While biological process (BP) gene ontology was used to construct network by ClueGO.

### 2.6 PPI network construction and cluster analysis

In order to find molecular interaction and possible functional module in targeted gene list of each miRNA, protein-protein interaction network was constructed by CluePedia [24], a powerful plugin for Cytoscape [25]. STRING [26] was used as an interaction resource and the confidence cut-off was set to 0.7 (high confidence). Molecular complex detection (MCODE) algorithm [27] was used to find clusters in PPI networks. Node density cut-off = 0 and node score cut-off = 0.2 were set as MCODE parameters.

## 3 Results

### 3.1 Data pre-processing

**Figure 1:**
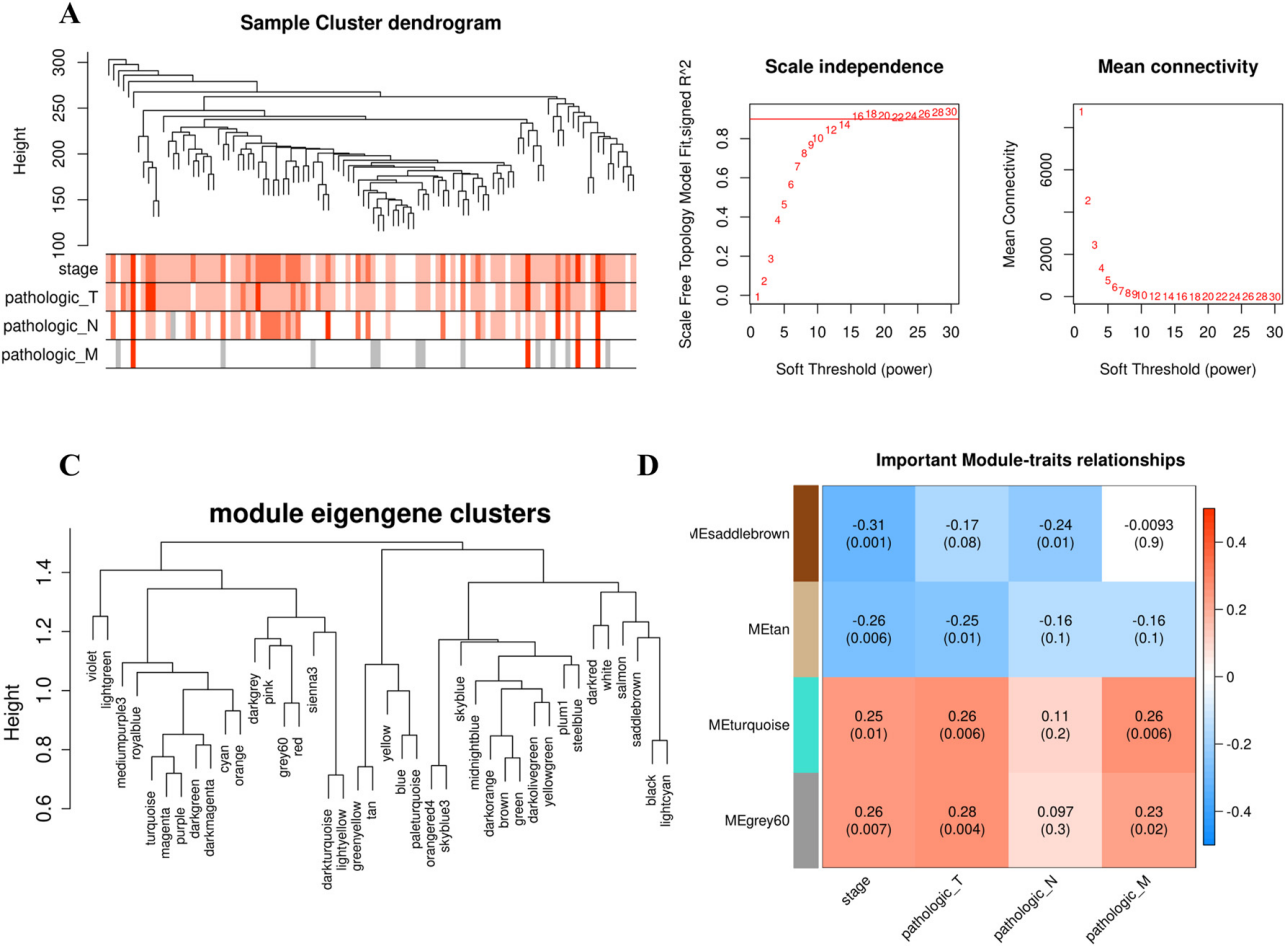
Dendrogram and heatmap represent distance between mRNA data samples and their corresponding stage and TNM data respectively (A) plots show scale free topology fitting *R*^2^ and mean connectivity of different powers. Power = 16 fulfills *R*^2^ > 0.9 and was chosen for network construction. (B) Dendrogram shows modules’ eigengenes and their distances. (C) Heatmap demonstrates important modules and correlations between their eigengenes and stage and TNM. *P*-values are also mentioned in parentheses (D).

After selecting treatment-naive patients and removing samples with missing mRNA, miRNA, or stage data, 106 out of 1098 TCGA-BRCA samples were included. Minimum, average, and maximum age of included patients was 30, 64, and 90 years respectively. Chosen data comprised 21 stage I, 58 stage II, 23 stage III, and 4 stage IV samples. Initial mRNA count matrix had 56602 Ensembl gene ID and after removing lowly expressed genes, it has 17463 ID in common with the merged tarbase and mirtarbase interaction data which had information of 20480 Ensembl gene ID. There was no missing data in count matrix and no outlier was seen in the data. Hierarchical clustering analysis and stage and TNM heatmap can be seen in Figure 1A.

### 3.2 Co-expression network construction and module detection

To construct co-expression network, correlation coefficients were raised to *β* = 16. As shown in Figure 1.B, it fitted scale-free topology by *R*^2^ = 0.915 and has mean connectivity = 8.11. Forty distinct modules were found in constructed network. Grey module which consists of unassigned genes was removed in further analysis. The smallest module (mediumpurple3) had 30 genes and the largest one (turquoise) had 1,539 genes. Figure 1c shows hierarchical clustering of modules’ eigengenes. Correlations between eigengenes and stage and TNM were calculated and among all modules, four modules had |correlation| > 0.2 with at least two of stage-related traits. Figure 1D shows stage_modules correlation heatmap and their corresponding *p*-values and top GO term and pathways for important modules can be seen in Table 1. Saddlebrown and tan modules have negative correlation with cancer progression and top results of their biological process gene ontology enrichment analysis were cilium movements and retinol metabolic process respectively. There were also two other important modules which show positive correlation with cancer progression. Turquoise module was one of them and mitochondrial processes such as mitochondrial translation and oxidative phosphorylation were among its top GO terms and pathways. Another one was grey60 module and it consisted of small group of genes that exist in cytoplasm and nucleoplasm.

**Table 1:** Pathways, and GO analysis of important modules. For each module, the first five functional terms, their resources, and corresponding FDR values are reported.

Modules	Term: resource (FDR)				
saddlebrown	BP: cilium movement (3.485150E-11)	BP: flagellated sperm motility (1.117428E-05)	BP: cilium movement involved in cell motility (3.521998E-05)	BP: cilium assembly (3.887220E-05)	CC: motile cilium (9.376820E-05)
tan	CC: extracellular region (4.142144E-05)	CC: plasma membrane (8.357566E-05)	CC: lipid particle (9.767895E-05)	BP: retinol metabolic process (0.000116)	KEGG: PPAR signaling pathway (0.000241)
turquoise	BP: mitochondrial translational elongation (5.559936E-019)	BP: mitochondrial translational termination (5.559936E-019)	BP: mRNA splicing, via spliceosome (2.402600E-11)	BP: NIK/NF-kappaB signaling (5.491643E-09)	BP: mitochondrial respiratory chain complex I assembly (1.003084E-08)
grey60	CC: cytosol (2.783334E-06)	CC: nucleoplasm (0.004703)	CC: shelterin complex (0.0236595)	CC: nuclear telomere cap complex (0.0236595)	MF: protein binding (0.0302450)

### 3.3 miRNA targets enrichment analysis

**Figure 2:**
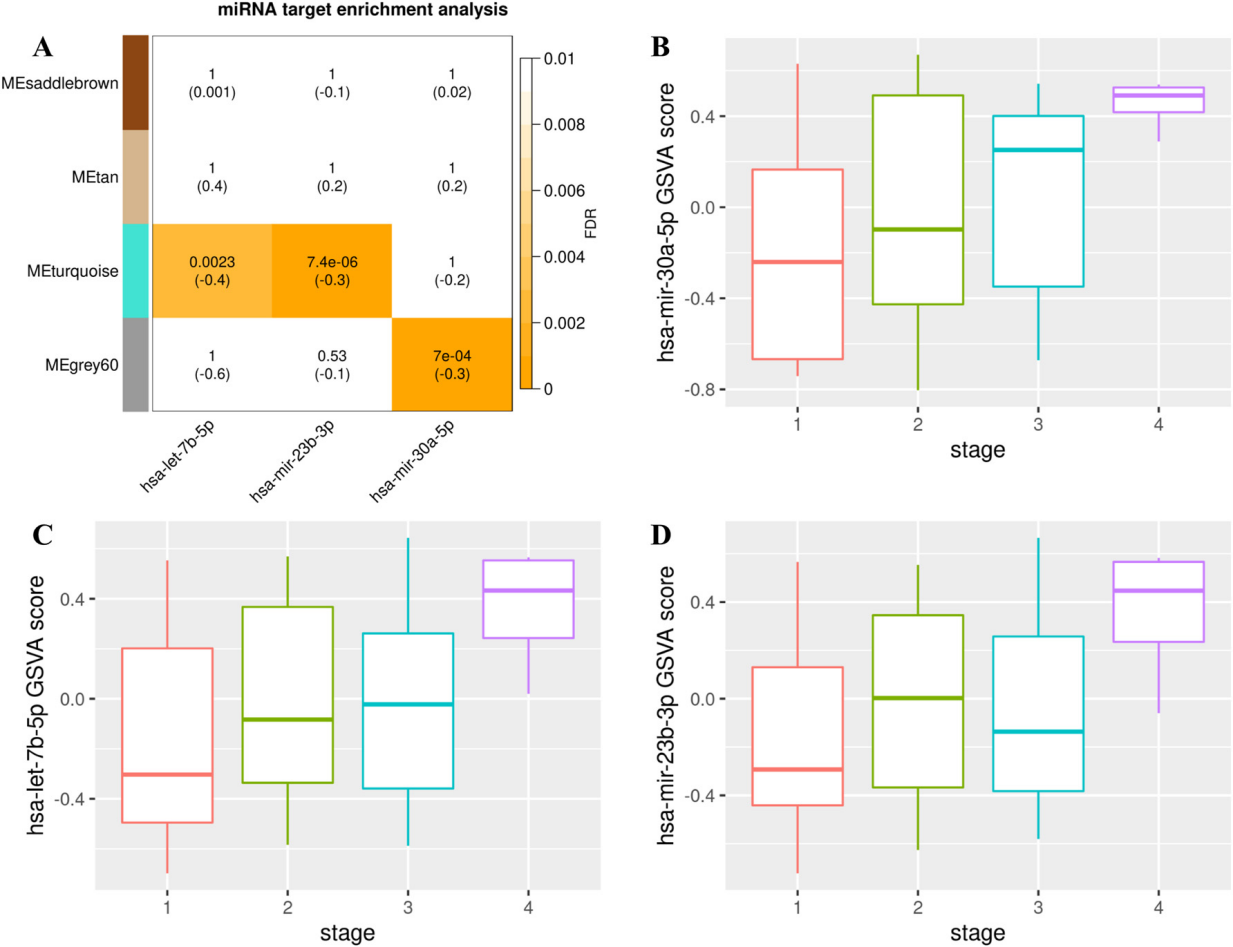
Heatmap shows important miRNAs and their target enrichment analysis result for important modules. Corresponding FDR values are shown and correlation between miRNA expression and modules’ eigengenes are mentioned in parentheses. (A) For each miRNA, its targeted genes were considered as a single gene list and GSVA score was calculated for them. Boxplots show GSVA scores of miRNAs’ targeted gene lists against stages. Coefficients and corresponding p-value regarding correlation between GSVA scores and stages have been mentioned above each plot (B–D).

After performing hypergeometric test and calculating false discovery rate (FDR), 29 miRNA passed FDR < 0.05 threshold. Moreover, correlations between normalized miRNA expression and modules’ eigengenes were calculated and correlation < −0.3 was used as the second level of threshold and three miRNA passed both threshold. Hsa-let-7b-5p and hsa-mir-23b-3p had 471 and 372 targets in the turquoise module respectively and hsa-mir-30a-5p had 28 targets in grey60 module. The turquoise module had 1,539 and the grey60 module had 92 genes and both modules were positively correlated with stage-related traits which shows suppression effect of mentioned miRNAs on the breast cancer progression. Important miRNAs’ target enrichment results and their correlation with important modules are depicted in Figure 2A.

### 3.4 Important miRNA targets GSVA score

For each important miRNA, their targets were considered as a gene set and gene set variation analysis (GSVA) score was calculated and compared among different stages. As shown in Figure 2B–D, targeted gene set of each important miRNA (hsa-mir-23a-3p, hsa-let-7b-5p, and hsa-mir30a-5p) had a higher GSVA score in higher stages.

### 3.5 Pathway and gene ontology enrichment analysis of miRNAs’ target gene sets

**Figure 3:**
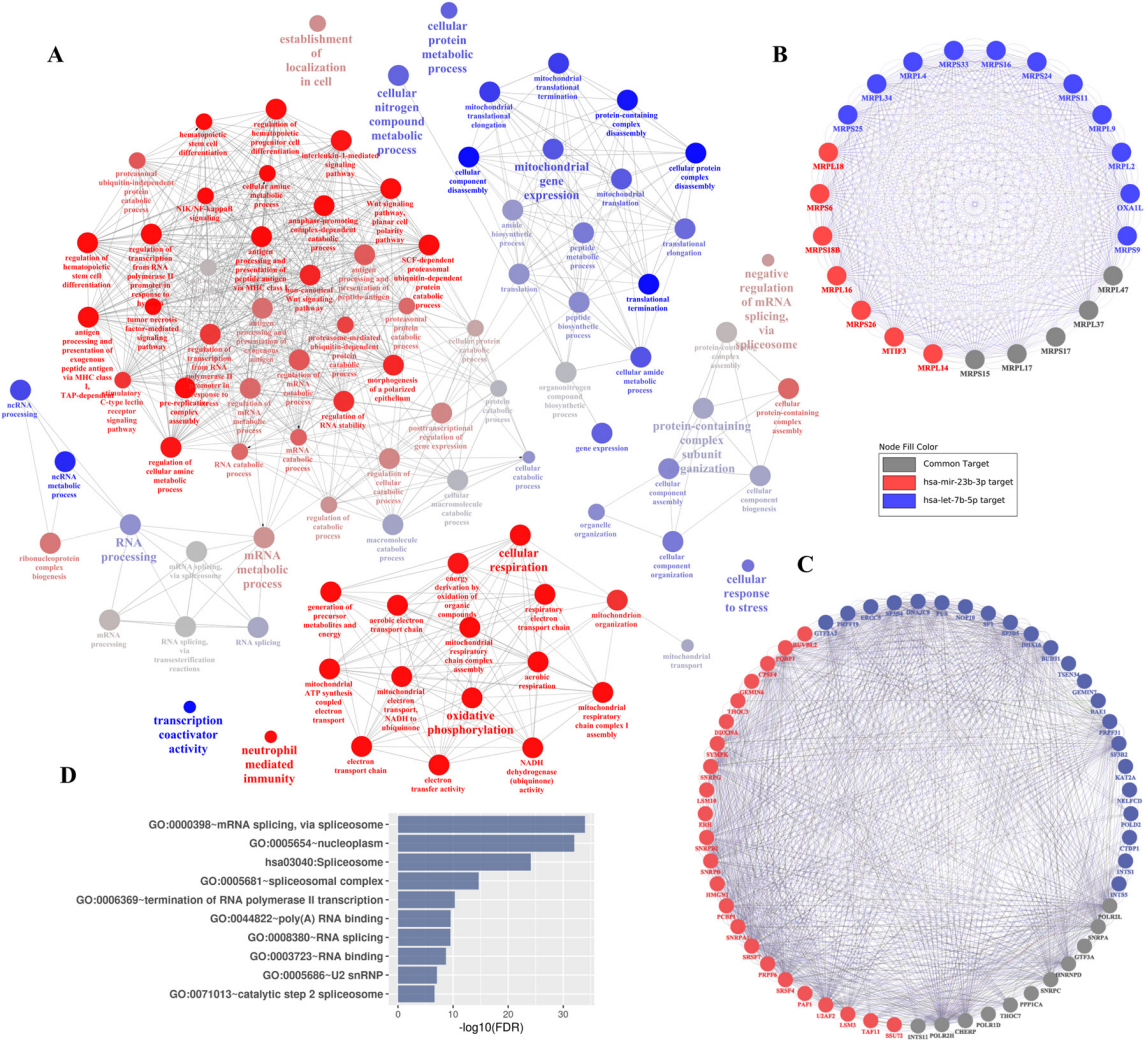
ClueGO result shows functional enrichment analysis for mir-23b and let-7b targets. Nodes show biological process GO terms with *p*-value < 0.05 and their colors indicate percentage of each miRNA’s targets. Blue nodes show terms consist of more than 60 % let-7b targeted genes and red nodes show terms with more than 60 % genes targeted by mir-23b. (A) MCODE first two modules are shown; blue and red nodes indicate let-7b and mir-23b targeted genes respectively and gray nodes are genes which are targeted by both. (B, C) DAVID pathway enrichment analysis result for second MCODE modules are sorted by log10(FDR) and plotted as bar-chart (D).

Because hsa-mir-23b-3p and hsa-let-7b-5p targets were significantly enriched in the same module (turquoise), their functional roles were investigated together and their targets were treated as a single gene list. As ClueGO result (Figure 3A) demonstrate, mir-23b and let-7b target related cellular activities as expected. Major cellular activities such as mRNA metabolism and processing, mitochondrial gene expression, and oxidative phosphorylation enhance in higher breast cancer stages and it can be due to reducing inhibitory effects of mir-23b and let7b. These miRNAs seem to have complementary roles in breast cancer. For instance, mRNA metabolism is mostly controlled by mir-23b and mRNA processing is affected by let-7b; or mitochondrial gene expression is regulated by let-7b and mir-23b regulates oxidative phosphorylation.

On the other hand, because hsa-mir-30a-5p targeted separated module (grey60), its functional roles were assessed individually. As represented in DAVID analysis (Table 2), targeted genes of mir-30a were mostly in cytosol and autophagy was a frequent term that had been seen in its functional analysis.

**Table 2:** DAVID analysis result of mir-30a targets. Terms and pathways which have FDR < 0.05 are shown. Their corresponding number and symbol of genes are also represented.

Term	FDR	Count	Genes
GO:0000421∼autophagosome membrane	0.001761	4	GABARAPL2, MAP1LC3B, CHMP1A, MAP1LC3B2
GO:0005829∼cytosol	0.001761	18	GABARAPL2, USP10, AKTIP, NIP7, SIAH1, CSNK2A2, MAP1LC3B2, MEAK7, ZNRF1, SLC7A5, MAP1LC3B, PSMD7, OGFOD1, POLR2C, CHMP1A, RANBP10, VPS35, NAE1
GO:0097352∼autophagosome maturation	0.006519	4	GABARAPL2, MAP1LC3B, CHMP1A, MAP1LC3B2
hsa04137:Mitophagy – animal	0.012069	4	GABARAPL2, MAP1LC3B, CSNK2A2, MAP1LC3B2
GO:0006995∼cellular response to nitrogen starvation	0.018318	3	GABARAPL2, MAP1LC3B, MAP1LC3B2
hsa04139:Mitophagy – yeast	0.021799	3	GABARAPL2, USP10, CSNK2A2
GO:0016236∼macroautophagy	0.026575	4	GABARAPL2, MAP1LC3B, CSNK2A2, MAP1LC3B2
GO:0000209∼protein polyubiquitination	0.034069	5	ZNRF1, PSMD7, CBFB, AKTIP, SIAH1

### 3.6 PPI network was constructed, and important modules were found

To investigate targets of mir-23b and let-7b holistically, PPI network was constructed by Cytoscape. Because targeted genes were originally co-expressed, it is possible to see different functional protein modules which work together in the same activity or even different genes which are subunits of one complex protein. To find these modules, MCODE was performed and two important modules with the highest scores were chosen. The first module which its MCODE score was 21.818 (Figure 3B), consists of 21 mithochondrial ribosomal proteins, mitochondrial translation initiation factor (MTIF1) and mitochondrial inner membrane protein OXA1L that interact with mitochondrial ribosomes and require for insertion of proteins to mitochondrial inner membrane. Second module with score = 18.182 is represented in Figure 3C. As DAVID analysis results indicated (Figure 3D), its genes are mostly involved in spliceosome and there are several genes related to transcription in this module such as several RNA polymerase subunits.

**Figure 4:**
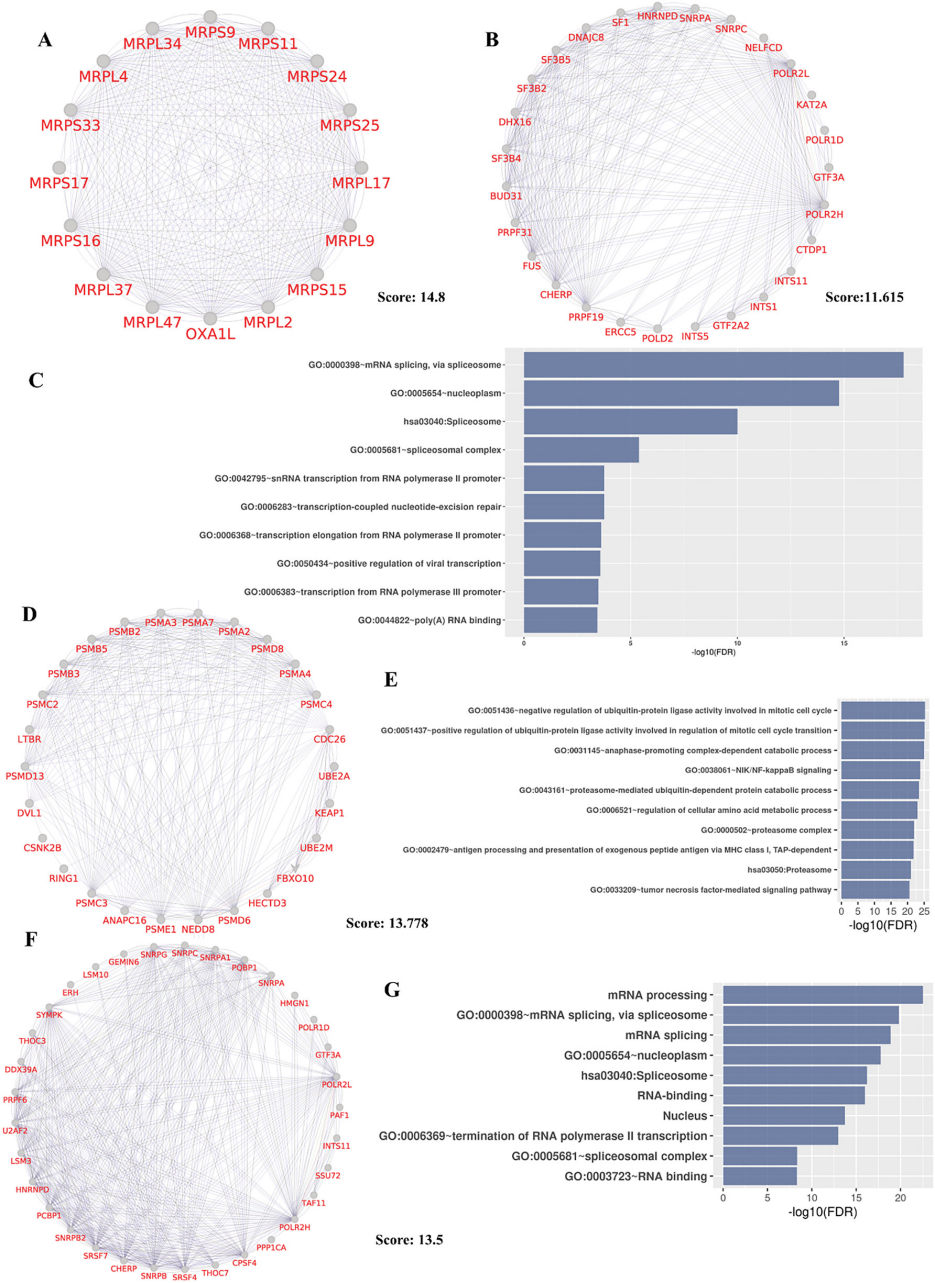
MCODE first and second modules of let-7b targets’ PPI networks are shown. (A, B) DAVID analysis result of second module is depicted as bar-chart (C). Proteins and their interaction, and functional analysis result of mir-23b targets’ first MCODE module (D, E) and the second module (F, G).

To examine mir-23b and let-7b targets in more detail, we also constructed separated PPI networks for each of them and run MCODE algorithm separately to find their important network’s clusters. For let-7b, the first module comprised mitochondrial ribosomal proteins and the second module consisted of spliceosomal proteins as same as modules in union PPI network of mir-23b and let-7b targets. Modules of let-7b targets’ PPI networks can be seen in Figure 4A and B and DAVID analysis result of second module is shown in Figure 4C. As shown in Figure 4D and E, first module of mir-23b targets mostly consists of proteosomal genes and its DAVID analysis result shows its genes mostly participate in ubiquitin activity in cell cycle and ubiquitin-dependent catabolism processes. Second module of mir-23b targets and their enrichment analysis are depicted in Figure 4F and G, and most of its nodes are spliceosomal protein and it indicates mRNA splicing as important target of both mir-23b and let-7b.

## 4 Discussion

Breast cancer is the most common cancer around the world and because of its complexity and heterogeneity, its molecular mechanism is not fully understood. Breast cancer cells can grow constantly, invade near tissues, and metastasis to other locations of the body. Better understanding of these complications at the molecular level can enhance our ability in breast cancer diagnosis and treatment.

In recent years, systems biology has revolutionized the way we were looking at biological systems and expanded our knowledge in molecular biology. System-level insights can help us to understand diseases and their molecular mechanisms better and also it can lead us to discovering new drugs and find new prognostic and diagnostic biomarkers. MiRNAs are among the most important components which play pivotal roles in health and diseases and because each one of them has several targets, systems biology approach is suitable to study their regulatory effects.

In this study we investigated TCGA-BRCA mRNA and miRNA data of treatment-naive patients to find important miRNAs in breast cancer progression. Due to regulatory effects of miRNAs on different targets, it’s expected to see common expression patterns in their targets. To unravel these types of similarities in expression pattern, first we used WGCNA to construct co-expression network and find modules of correlated genes. Second we chose four important modules whose representative values (eigengenes) have statistically important correlation with stage; two of them had negative correlation and the other two modules had positive correlation with stage. Then we examine possible regulatory effects of miRNAs on those important modules by target enrichment analysis. We used hypergeometric test to do so and adjusted p-value and negative correlations between miRNAs and modules’ eigengenes were used to find important miRNAs with possible regulatory effects on modules. We found three miRNAs by applying these criteria which have both significantly enriched targets and correlation with one of the four important modules. All of those three miRNAs targeted modules with positive correlations with stage.

### 4.1 Hsa-mir-30a-5p

One of the important miRNAs we found was hsa-mir-30a-5p and pathway enrichment analysis of its target showed autophagy as an important term. Several studies have shown mir-30a is a tumor suppressor miRNA in breast cancer and revealed its suppression effect on progression and metastasis 28], [29], [30], [31. Also its higher level of expression has been shown to be associated with better overall survival of patients [32]. Moreover there are also several studies that show mir-30a has an inhibitory effect on autophagy in different types of cancers including breast cancer [33, 34]. Most of these studies focused on beclin-1 as autophagy-related target of mir-30a, but here our results showed that the inhibitory effect of mir-30a goes behind beclin-1 and there are several autophagy-related genes which are targeted by mir-30a in breast cancer. Autophagy is a crucial process in different types of cancer and it plays an important role in breast cancer tumorigenicity [35]. There are also several studies indicated that it can have both anti- and pro-metastatic effects on breast cancer cells. One of the possible explanations is autophagy can help unattached cells to survive before dormancy in new environments [36] and it can explain why the lower expression of mir-30a is associated with growth in unattached breast cancer cells [37].

### 4.2 Hsa-let-7b-5p

Hsa-let-7b-5p was another miRNA that we found to be important in breast cancer progression. Let-7 microRNA family have been shown to be dysregulated in several malignancy in both cancerous tissues and blood sample of patients [38]. This family have been indicated to regulate JAK-STAT3 pathway via STAT3 targeting, and MYC pathway by regulating expression of c-MYC. Also there are evidences which shows the regulatory role of let-7 family on breast cancer metastasis and stemness mostly by targeting oncogenes such as RAS, HMGA2 [39]. Regarding to let-7b, a member of let-7 family, there is also an *in vivo* evidence of their downregulation in breast cancer [40]. Dysregulation of let-7b have been also shown *in vitro*, and it’s role in the apoptosis process has been indicated by breast cancer cell line transfection [41]. Moreover, a study indicated the effect of let-7b on cell growth and metastasis through reprogramming aerobic glycolysis via modulation of Hexokinase 2 (HK2) [42]. This had been followed by another evidence which indicated the role of metabolic reprogramming via let-7 family to be associated with tumor progression and autophagy [43].

Here we have found the targets of let-7b had been enriched in one of the important module in the breast cancer co-expression network (turquoise module) which had positive correlation with progression traits. Pathway enrichment analysis showed mitochondrial gene expression is in this module and targeted by let-7b. Moreover PPI network analysis of let-7b targets in the turquoise module, indicated this process maybe targeted mostly by targeting mitochondrial ribosomal gene expression. Localization of precursor and mature form of let-7 family microRNA have been indicated previously by *in situ* hybridization [44]. our results suggested metabolic reprogramming by let-7b could be due to upstream modulation of mitochondrial genes’ expression indeed by controlling regulation of mitochondrial ribosomal proteins. Also mitochondrial ribosomal genes belonged to breast cancer progression related module (turquoise). This suggest the importance of mitochondrial ribosome in breast cancer cell growth and metastasis. There are studies that showed the importance of mitochondrial ribosomes in breast cancer and their subunits are differentially expressed in this disease [45, 46]. Here we found this dysregulation maybe caused by let-7b.

### 4.3 Hsa-mir-23b-3p

Also we found hsa-mir-23b-3p as another important miRNA and together with let-7b, they have significant number of targets in the same module. Mir-23b have been shown to by dysregulated in several malignancies. Mir-23b has been shown to have dual role in different cancer types and evidences have indicated both anti-tumor effects and tumor promoting effects for this microRNA [47]. Also in breast cancer, there are several studies show mir-23b dysregulation but there is also duality of its roles in breast cancer formation and progression. Some studies emphasized on oncogenic effects of mir-23b and their positive effect on breast cancer progression. For instance, knockout of mir-23b and mir-27b have been shown to reduce cell growth in breast cancer cell line (MCF7) but had no effect on migratory behavior of breast cancer cell line [48]. Another study indicated elevation in mir-23b expression in malignant breast cancer tissue comparing to normal tissue; but decreasing in higher stages comparing to lower stages [49]. Other studies addressed their tumor-suppressing roles especially on progression and metastasis. Using mir-23b transfection in breast cancer cell line, one study have shown regulatory role of mir-23b on several cytoskeletal genes’ expression which cause cytoskeleton remodeling and increase motility of cancer cells [50]. Also another evidence indicated mir-23b stabilized epithelial phenotype and reverse Epithelial Mesenchymal Transition (EMT) which is an important behavior in metastasis [51].

Here we found mir-23b may have tumor-suppressing role in breast cancer and inhibits progression and metastasis. We have found mir-23b targets have been enriched in turquoise module and they were mostly involved in oxidative phosphorylation process and NF-KB signaling pathway. Previous study showed suppression of mir-23b expression by c-myc can change metabolism program and enhances glutamine metabolism. Considering converting glucose to lactate in anaerobic metabolism in cancer cells (Warburg effect), glutamine metabolism is pivotal to maintaining mitochondrial metabolism in cancer cells [52]. This phenomenon (glutamine metabolism) have been shown to be an important behavior in breast cancer cells like other malignancy [53]. Here we have shown oxidative phosphorylation in mitochondria is correlated to breast cancer progression and mir-23b maybe the key regulatory element in this process. Also there are a line of evidences that have shown NF-KB pathway can change the expression of mir-23b by increasing its expression [54, 55]. Interestingly our result indicated the regulatory effect of mir-23b on NF-KB pathway. This have been also demonstrated in other studies [56, 57]. This would be suggesting feedback loop between the expression of mir-23b and NF-KB pathway. Further study is necessary to investigate relationship between mir-23b and NF-KB to unravel their regulatory effects on each other in breast cancer.

Also our results show that let-7b and mir-23b affect mRNA metabolism and processing such as mRNA splicing in breast cancer. Spliceosomes play important roles in breast cancer and their components have been shown to be related to breast cancer patients’ survival [58]. To our knowledge, no study have demonstrated complementary role of let-7b and mir-23b in breast cancer. Also we showed several new processes which are targeted by these two miRNAs in breast cancer.

In summary, we identified mir-30a, mir-23b, and let-7b as important miRNAs related to breast cancer progression by enrichment analysis of WGCNA modules. They regulate expression of not just few specific genes but two progression-related modules in breast cancer which may contain even several hundreds of genes. We found mir-23b and let-7b targeted module contains metabolic-related genes and mir-30a targets several autophagy-related genes. Our finding revealed new mechanisms of these miRNAs in breast cancer and their possible roles in breast cancer progression.
